# Postsynaptic Congenital Myasthenic Syndrome Mimicking Limb–Girdle Muscular Dystrophy Associated with an Alternatively Spliced Exon in CHRNB1: A Case Report and Literature Review

**DOI:** 10.3390/children13060841

**Published:** 2026-06-22

**Authors:** Wen-Kan Feng, Kun-Long Hung, Ting-Hao Wang

**Affiliations:** 1 Department of Pediatrics, Sijhih Cathay General Hospital, New Taipei City 22174, Taiwan; fengkv@gmail.com (W.-K.F.); james1141wth@hotmail.com (T.-H.W.); 2 Department of Pediatrics, Fu Jen Catholic University Hospital, New Taipei City 24352, Taiwan; 3 School of Medicine, Fu Jen Catholic University, New Taipei City 24205, Taiwan

**Keywords:** congenital myasthenic syndrome, limb–girdle muscular dystrophy, neuromuscular junction, CHRNB1, slow-channel syndrome

## Abstract

**Highlights:**

**What are the main findings?**

- An adolescent male presented with a limb–girdle muscular dystrophy–like phenotype was finally diagnosed as postnatal congenital myasthenic syndrome after genetic identification of a hetrogyzous CHRNB1 mutation by WES.
- Clinical improvement was observed after adjusting the therapeutic regimen.

**What is the implication of the main finding?**

- Diagnostic challenges exist in patients with atypical congenital mysthenic syndrome presentations.
- The importance of molecular genetic testing in guiding accurate diagnosis and targeted therapy is demonstrated.

**Abstract:**

Fatigue and muscle wasting are common clinical manifestations of inherited and acquired neuromuscular disorders, including peripheral neuropathies, neuromuscular junction disorders, and myopathies. These conditions encompass a wide disease spectrum with variable prognoses, making accurate diagnosis essential for appropriate management. Congenital myasthenic syndromes (CMSs) are rare, inherited disorders characterized by impaired neuromuscular transmission. Although symptoms often begin in infancy or early childhood, later onset during adolescence or adulthood is increasingly recognized. Clinical phenotypes vary according to the underlying molecular defect, but fatigable weakness predominantly affecting axial and proximal limb muscles is a hallmark feature. We report an adolescent male who developed progressive proximal muscle weakness and wasting over several years, resulting in significant functional impairment. Initial evaluation suggested limb–girdle muscular dystrophy. However, comprehensive investigations, including whole-exome sequencing, identified a heterozygous CHRNB1 mutation consistent with postsynaptic CMS. Targeted pharmacological therapy led to clinical improvement. This case highlights the importance of considering CMS in patients presenting with limb–girdle weakness and underscores the value of genetic testing in establishing an accurate diagnosis and guiding treatment.

## 1. Introduction

Congenital myasthenic syndromes (CMSs) are inherited disorders of the neuromuscular junction characterized by fatigable muscle weakness with onset ranging from infancy to adulthood. In contrast, acetylcholine receptor (AChR) deficiency typically presents at birth or during early infancy. CMSs are frequently misdiagnosed as congenital muscular dystrophies or primary myopathies [1,2]. Certain types of CMSs (especially limb–girdle type) are misdiagnosed as limb–girdle muscular dystrophy or congenital myopathy leading to delayed diagnosis and inappropriate treatment. Previous studies have reported misdiagnosis rates of up to 80% in pediatric patients [3].

CMSs can be classified according to the site of dysfunction—presynaptic, synaptic, or postsynaptic—or by the underlying pathogenic mechanism, such as AChR deficiency or abnormal channel kinetics [4]. Recognition of CMSs is clinically important because several subtypes are amenable to targeted pharmacological therapy [5]. We herein report an adolescent male presenting with symptoms mimicking limb–girdle muscular dystrophy. Yet after comprehensive investigations, including electrophysiological study and whole-exome sequencing, a heterozygous CHRNB1 mutation was identified, consistent with the diagnosis of postsynaptic CMS. Clinical improvement was observed after targeting the therapeutic regimen.

## 2. Case Report

A 16-year-old male presented with a three-year history of gradually progressive muscle weakness. He had been previously healthy and developmentally normal. The weakness initially involved the lower limbs and later affected the upper limbs, resulting in exercise intolerance and increasing difficulty climbing stairs. Progressive wasting of the shoulder girdle, buttocks, and thighs was noted.

**Figure 1:**
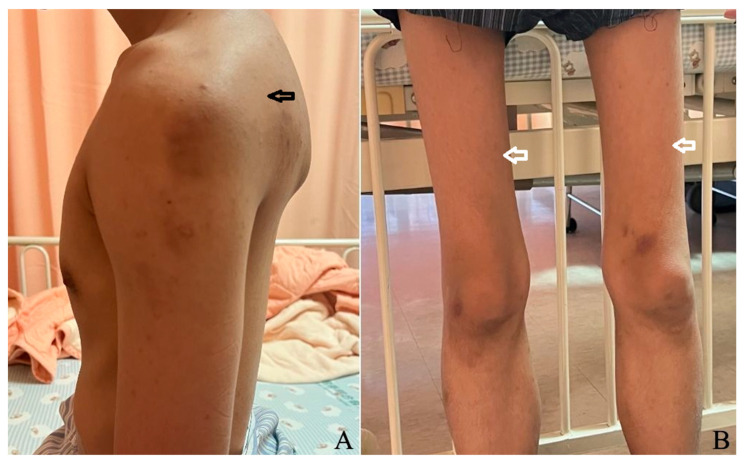
Clinical appearance of the patient showing marked wasting of the shoulder girdle ((**A**), black arrow) with scapular winging and atrophy of the buttock and thigh muscles ((**B**), white arrow).

On physical examination, the patient appeared tall and thin and walked with a slow, limping gait. Marked wasting of the shoulder girdle with scapular winging and atrophy of the buttock and thigh muscles were observed (Figure 1). He was unable to raise his arms above shoulder level. He walked with a waddling gait and Gowers’ sign was positive. His muscle power decreased to grade 3 in both proximal limbs. Deep tendon reflexes were also found to be less reactive. Based on the clinical presentation, limb–girdle muscular dystrophy was initially suspected.

Laboratory investigations revealed mildly elevated muscle enzymes, including creatine phosphokinase (CPK) at 517 IU/L (reference range: 30–223 IU/L), CPK-MB at 31.2 ng/mL (0.6–6.3 ng/mL), and myoglobin at 153.4 ng/mL (0.0–105.7 ng/mL). Nerve conduction studies showed normal latency and conduction velocity in the bilateral median nerves and ulnar nerves. Repetitive nerve stimulation, sensory nerve conduction studies, F-waves, and H-reflexes were within normal limits. Needle electromyography (model UltraPro S 100, Alpine Biomed ApS, Skovlunde, Denmark, 2018) demonstrated reduced interference patterns in the right deltoid, pectoralis major, tibialis anterior, and quadriceps femoris muscles.

Magnetic resonance imaging (performed on PHILIPS MR Systems Ingenia Ambition X machine with a nominal main magnetic field (B0) 1.5 T) of the lumbosacral spine showed no spinal cord or vertebral abnormalities; however, atrophy of the right gluteus and pectineus muscles was noted. Muscle biopsy (performed by a plastic surgeon under local anesthesia) from the left thigh revealed focal fiber atrophy at the periphery of muscle fascicles with few focal perivascular and perimysial lymphocytic infiltration. Immunohistochemistry demonstrated CD3-positive lymphoid cells, while CD20 staining was negative. Masson’s trichrome staining showed focal interstitial fibrosis. These findings were nonspecific and could not exclude a myopathic disorder or muscular dystrophy.

**Figure 2:**
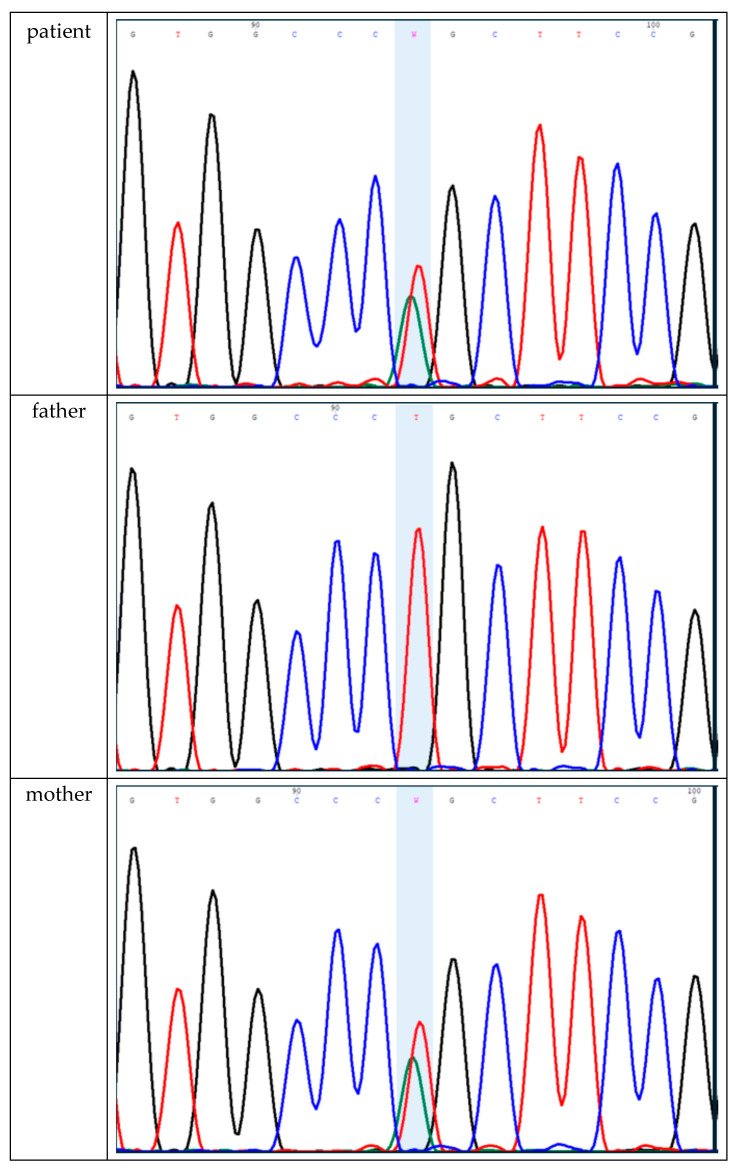
Sanger sequencing identified that the CHRNB1 NM_000747.3:c.1286T>A mutation point in the patient matched that of his mother.

Whole-exome sequencing (WES) with mitochondrial DNA analysis identified a heterozygous missense mutation in the CHRNB1 gene (chr17:7455862 NM_000747.3:c.1286T>A p.Leu429Gln) (Table 1). The mean depth of WES data was 127× and the coverage of 20× was over 98%. The prioritization of variants was based on patient phenotype by using Geneyx interpretation software 6.0. According to the ACMG classification, the variant is VOUS (PM2 and PP3). No pathogenic variants were detected in 39 genes associated with limb–girdle muscular dystrophy. A diagnosis of postsynaptic congenital myasthenic syndrome was therefore established. Sanger sequencing of the patient and his parents was determined. The CHRNB1 c.1286T>A/WT heterozygous missense mutation is inherited from his mother (Figure 2).

The patient was treated with fluoxetine 20 mg once daily and a β2-adrenergic receptor agonist (Albuterol) 25 mg twice daily, with subsequent clinical improvement. The strength and motion of his upper limbs were restored and muscle power of both thighs gradually improved. About one year after receiving the regimen, the strength of both the upper and lower limbs was almost restored. The motion of the upper limbs was observed to be normal and he can stand up from a squatting position without support. According to the Medical Research Council (MRC) scale, muscle strength was grade 5 in the upper limbs, and grade 4 in the lower limbs is. However, wasting of both thigh muscles has not yet recovered.

## 3. Discussion

In cases of myasthenia gravis (MG), autoimmune myasthenia gravis with autoantibodies targeting the acetylcholine receptor (AChR) is dominant in 85% of patients. Genomic studies highlighted the association of genes encoding AChR subunits (CHRNA1—cholinergic receptor nicotinic alpha 1 subunit and CHRNB1—cholinergic receptor nicotinic beta 1 subunit) that lead to a diagnosis of congenital myasthenia syndromes (CMSs) [6]. Congenital myasthenia syndromes are clinically and genetically heterogeneous but treatable diseases. To date, mutations in more than 35 genes have been associated with CMSs [7]. Postsynaptic CMS represents the most common subtype and primarily involves abnormalities of the muscle AChR. Approximately 75% of CMS cases are postsynaptic with the majority caused by AChR-related defects, compared to 15% synaptic and 10% presynaptic [4,5].

The postsynaptic CMS is triggered by mutations in the genes encoding different subunits of the muscle nicotinic AChR. The causes of the postsynaptic CMS group are specific AChR kinetic abnormalities that result in the abnormally brief (fast-channel syndrome) or abnormally prolonged (slow-channel syndrome) AChR channel openings with corresponding effects on endplate current duration and deleterious effect on neuromuscular transmission [8,9].

Endplate AChR deficiency caused by pathogenic variants in CHRNA1, CHRNB1, CHRND, and CHRNE and leading to reduced AChR expression as slow-channel syndrome (SCCMS) or fast-channel syndrome (FCCMS), has been repeatedly reported since 1996 [10].

In the neuromuscular junction, the release of acetylcholine (ACh)-containing vesicles into the synapse from the presynaptic terminal, activates the post synaptic acetylcholine receptor (AChR), and evokes a local depolarization and an endplate potential (EPP), which is proportional to the amount of ACh binding to the AChR. EPP triggers the NaV1.4-type voltage-gated sodium channels leading to local depolarization of the muscle membrane. All CMSs have been linked to a physiologic defect that leads to a subthreshold EPP that fails to trigger the NaV1.4, or to a defect in the NaV1.4, both of which cause deleterious effects on neuromuscular transmission [8].

The CHRNB1 gene encodes the β-subunit of the AChR, a pentameric ligand-gated ion channel that undergoes conformational changes upon acetylcholine binding, allowing cation influx across the postsynaptic membrane. CHRNB1 mutations often cause postsynaptic CMS by reducing the number of functional nicotinic acetylcholine receptors (AChR) at the motor endplate. Mutations in CHRNB1 have been increasingly recognized as a cause of slow-channel congenital myasthenic syndrome [5,11]. These mutations lead to prolonged channel opening, excessive cation influx, and subsequent endplate myopathy. Prolonged openings of AChR ion channels increase the intracellular Na+ concentration and depolarize the resting membrane potential, which reduces the amplitude of an endplate potential (EPP) and makes it difficult for the muscle sodium channel (NaV1.4) difficult to sense the EPP. As AChR is a cation-non-selective ion channel, prolonged openings of AChR Ca2+ channels allow excessive influx of Ca2+ ions, which causes endplate myopathy. Prolonged openings of AChR also desensitize AChR. AChR is physiologically desensitized by prolonged existence of Ach. Desensitized AChR does not respond to ACh and cannot generate EPP anymore. In the desensitized state, the two agonist-binding sites between the α-δ and α-ε subunits of AChR are rotated counterclockwise perpendicular to the membrane, and the structure of the extracellular end of the M4 helix of the α subunit that interfaces with the subunit becomes significantly different [12].

CHRNB1 mutations are typically inherited in an autosomal recessive manner; however, certain variants, particularly those associated with slow-channel congenital myasthenic syndrome, have been reported to exhibit autosomal dominant inheritance. In the present case, the heterozygous CHRNB1 missense variant c.1286T>A (p.Leu429Gln) was found in both the proband and his mother, suggesting autosomal dominant transmission. Notably, the mother is clinically unaffected and has remained healthy throughout her life, indicating possible incomplete penetrance of this variant. The patient had an earlier age onset and more severe initial symptoms due to CHRNA1, CHRNB1, and CHRND mutations. CHRNA1, CHRNB1, and CHRND mutations tend to result in a more severe phenotype than those with underlying CHRNE variants, with typical manifestations of muscle weakness, including ocular, limb, and bulbar symptoms [13].

Clinical phenotypes of slow-channel CMSs are variable, with onsets ranging from infancy to adulthood. Most CMS patients develop the disease before 2 years of age, but, in some patients, symptoms develop immediately after birth and temporarily subside, resembling muscular dystrophy. Adult-onset patients tend to have mild phenotypes. In contrast to MG, diurnal fluctuation of muscle strength and muscle fatigue are not always observed in CMS [10]. Weakness may be generalized or selectively involve scapular, cervical, and proximal limb muscles, with or without ocular involvement. Serum CK levels are normal in most CMS groups. However, serum CK levels may be elevated ~1.5 times the upper limit of normal in endplate myopathies in SCCMS [7]. Muscle biopsy findings are often nonspecific and may demonstrate myopathic changes, including fiber size variation and fibrosis [1].

CMS may be a treatable disorder. CMS treatment depends on the underlying molecular mechanism. Treatment usually involves acetylcholinesterase inhibitors (such as pyridostigmine) to increase acetylcholine at the synapse. Acetylcholinesterase inhibitors are beneficial in some CMS subtypes but may be ineffective or harmful in slow-channel CMSs. Unlike autoimmune myasthenia gravis, immunosuppressant and immunomodulatory treatments do not play a role in the management of CMS, which is determined on a rational basis depending on the postulated underlying defects in neuromuscular transmission [8]. In the slow-channel syndrome, excess stimulation results from the prolonged burst duration of the AChR.

Fluoxetine and quinidine act as open-channel blockers of the AChR, reducing excessive cation flow and limiting endplate damage. Both are long-lived open-channel blockers of the AChR with the capacity to block it in its open state and shorten the duration of otherwise prolonged synaptic currents. β2-adrenergic receptor agonists, such as salbutamol or ephedrine, may further improve neuromuscular transmission by stabilizing postsynaptic structures and enhancing AChR clustering [2,3,11,14,15]. Patients receiving oral salbutamol did report significant and sustained improvement in fatigue after 6 and 12 months of treatment [16,17].

Although fluoxetine can produce marked clinical improvement in slow-channel CMS, potential adverse effects—including psychiatric symptoms and QT interval prolongation—require careful monitoring. Quinidine is associated with cardiac conduction abnormalities and should be used cautiously. As shown in the present case, combined therapy with fluoxetine and a β2-adrenergic receptor agonist resulted in functional improvement [11,18].

## 4. Conclusions

Genetic classification of the different CMS cohorts reveals that the majority of CMS patients have mutations in genes expressing post-synaptic endplate proteins [19]. With the widespread availability of next-generation sequencing, the number of genetically confirmed CMS cases has increased substantially [20,21]. CHRNB1 mutations often cause a postsynaptic CMS by reducing the number of functional nicotinic acetylcholine receptors (AChR) at the motor endplate. CMS is usually inherited in an autosomal recessive pattern, yet some mutations (e.g., slow-channel) can exhibit autosomal dominant inheritance. Although CMS classically presents in infancy, later onset during childhood or adulthood is increasingly recognized. Clinical phenotypes are heterogeneous, but fatigable weakness of axial and proximal limb muscles remains a key feature. It is stressed that the possibility of CMS should be kept in mind when facing a case of late-manifesting myasthenic pictures [11,22].

Accurate molecular diagnosis is essential, as treatment responses vary according to the genetic subtype. An agent that provides benefit in one CMS subtype can be harmful in another [23]. Thus, it is important to obtain an early genetic diagnosis, since inappropriate therapy may exacerbate symptoms [24]. Through this case, we emphasize the importance of considering CMS in patients presenting with limb–girdle weakness and highlights the critical role of genetic testing in guiding subtype-specific treatment.

## Figures and Tables

**Table 1:** Whole exome sequencing (WES) analysis of the patient.

Gene	Chr. Position	Variant Type	Exon/Intron Number	Genotype	Variant Information /Annotation	
FGFR3	chr4:1805794 NM_000142.5:c.1690C>G p.Arg564Gly	Missense	13/18	Heterozygote	Mode of Inheritance	AD, AR
					ClinVar	NA
					ACMG	Uncertain Significance
CHRNB1	chr17:7455862 NM_000747.3:c.1286T>A p.Leu429Gln	Missense	10/11	Heterozygote	Mode of Inheritance	AD, AR
					ClinVar	NA
					ACMG	Uncertain Significance

## Data Availability

Data sharing is not applicable to this article as no datasets were generated or analyzed.
